# Advances in Adjuvanted Rabies Vaccines

**DOI:** 10.3390/vaccines14020132

**Published:** 2026-01-28

**Authors:** Yutian Wang, Hongliang Sun, Yehong Wu

**Affiliations:** 1 Changchun Institute of Biological Products Co., Ltd., Changchun 130012, China; yutianwang0225@163.com; 2 State Key Laboratory of Novel Vaccines for Emerging Infectious Diseases, China National Biotec Group Co., Ltd., Beijing 100024, China

**Keywords:** human rabies vaccine, adjuvant, vaccination schedule, immune response

## Abstract

Rabies is an acute and fatal zoonotic disease caused by the rabies virus, responsible for approximately 59,000 deaths worldwide each year. Once clinical symptoms manifest, the case fatality rate approaches 100%. Vaccination remains the only effective strategy for prevention and control. Currently, human rabies vaccines approved by regulatory authorities such as the U.S. Food and Drug Administration (FDA), and the China National Medical Products Administration (NMPA) are all inactivated, adjuvant-free formulations. These vaccines are associated with several limitations, including weak immunogenicity, delayed induction of neutralizing antibodies, complex immunization schedules, and poor patient compliance. Adjuvants, as nonspecific immunoenhancers, can potentiate the immune response even at low antigen doses and reduce the number of required doses, offering a promising approach to overcome the aforementioned challenges. This article reviews recent advances in adjuvants suitable for rabies vaccines and discusses the key challenges currently faced in the development of adjuvanted rabies vaccines.

## 1. Introduction

Rabies is an acute, fatal viral encephalomyelitis caused by infection with the rabies virus (scientific name: *Rabies lyssavirus*), which belongs to the genus *Lyssavirus* within the family *Rhabdoviridae*. The virus typically enters the human body through broken skin or mucous membranes via the saliva of an infected host animal, usually through bites or scratches [1]. As a classic zoonotic disease, it continues to pose a serious worldwide public health threat. According to World Health Organization (WHO) data, rabies causes approximately 59,000 deaths globally each year—averaging about one death every nine minutes [2]. This mortality exhibits a highly uneven geographical distribution: while rabies is endemic in over 150 countries and regions, the vast majority of deaths are concentrated in developing nations across Asia and Africa [3]. A major reason for this disparity lies in weaknesses within the canine rabies control chain. In many low- and middle-income regions, low dog vaccination coverage, inadequate management of stray animals, limited public access to post-exposure prophylaxis (PEP), and its substantial financial burden collectively sustain viral circulation in the primary reservoir (dogs), ultimately leading to spillover into human populations [4].

The natural course of rabies is marked by considerable uncertainty. The incubation period is highly variable, typically on the order of 4 to 6 weeks, but can range from less than 10 days to more than 6 years [5]. Its duration is influenced mainly by factors such as the site of viral entry (bites to the head and face are associated with shorter incubation periods) and viral load [6]. Following the prodromal stage, which is initially nonspecific and consists of signs and symptoms compatible with a “flu-like illness,” such as fever and general malaise, the disease enters an acute neurological phase that is invariably fatal. This phase may present as furious rabies or as a paralytic form termed “dumb rabies”. Clinical presentation includes anxiety, agitation, hallucinations, cranial nerve deficits, dysphagia, hypersalivation, paralysis, and classic symptoms of paresthesia at the site of bite exposure and hydrophobia or aerophobia manifesting as phobic pharyngeal spasms. The clinical course is acute, with death usually ensuing within days once clinical signs are present [5]. To date, the case fatality rate following the onset of clinical symptoms approaches 100%, making rabies one of the most lethal infectious diseases globally [7].

Confronting this fatal yet preventable disease, timely and scientifically guided vaccination remains the only effective countermeasure. Prevention strategies are classified into two categories according to exposure risk: pre-exposure prophylaxis (PrEP) and PEP. PrEP is recommended for individuals whose occupational or environmental circumstances entail a sustained, frequent, or elevated risk of exposure, such as veterinarians, laboratory personnel working with the virus, children residing in rabies-endemic regions, and travelers to high-risk areas. PEP is stratified into three grades (I, II, and III) based on the nature of contact with a potentially rabid animal. Each grade corresponds to a distinct management protocol, which may include no intervention, immediate vaccination only, or vaccination combined with rabies immunoglobulin administration [8].

Upon entering the host, the rabies virus undergoes initial, slow replication within local tissues such as muscle cells [9]. Using its surface glycoprotein (G protein), the virus binds to specific receptors on nerve endings at the neuromuscular junction, initiating neuronal infection. Once inside a nerve terminal, the virus is enveloped by the cellular membrane to form an endosome. It then exploits the neuron’s own retrograde axonal transport system—normally responsible for carrying nutrient signals—and travels along nerve fibers toward the spinal cord and brain at a rate of approximately 50–100 mm per day. Once the rabies virus reaches the central nervous system and initiates replication, it rapidly disseminates and invades neural tissues [10]. Consequently, the critical objective of post-exposure immunization is to elicit the production of effective neutralizing antibodies prior to the virus entering the neural circuitry and accessing the central nervous system, thereby clearing extracellular virus particles and halting its progression toward further neural infection [11].

Currently, the routinely administered rabies vaccines are primarily cell culture-based purified vaccines that do not contain adjuvants. The traditional multi-dose immunization schedule is relatively lengthy, which often results in suboptimal patient compliance [12]. Adjuvants serve as nonspecific immunoenhancers that, while lacking antigenicity themselves, can substantially amplify the body’s immune response to a vaccine. By enabling lower antigen doses to induce protective cellular or humoral immunity, adjuvants represent a promising approach to improving vaccine efficacy [13]. In this review, we outline currently approved rabies vaccines and immunization regimens, summarize recent advances in adjuvants suitable for rabies vaccines, and discuss key challenges in the development of adjuvanted rabies vaccines.

This review was conducted based on a systematic literature search. The search was performed using platforms such as PubMed, Web of Science, Google Scholar, and China National Knowledge Infrastructure (CNKI), covering primarily the period from 2016 to 2025, with key earlier publications also included for context. Relevant English keywords and their combinations included “rabies vaccine,” “adjuvant,” “MPLA,” “CpG,” “PIKA,” “QS-21,” “mRNA vaccine,” and “post-exposure prophylaxis.” Corresponding Chinese keywords, such as “rabies”, “vaccine”, and “adjuvant”, were also utilized. The selection of literature adhered to the following criteria: (1) Studies focusing directly on the mechanisms, development, or evaluation of novel adjuvants or new technological platforms (e.g., mRNA vaccines) for rabies vaccines; (2) Priority given to original research articles, authoritative reviews, and official guidelines from organizations like the WHO and the Chinese Center for Disease Control and Prevention; (3) Emphasis on recent, high-impact literature published within the last five years. Through screening titles, abstracts, and subsequent full-text review, 60 references most pertinent to the core themes of this review were ultimately included to serve as the foundation for the discussion.

## 2. Currently Approved Rabies Vaccines

Currently approved human rabies vaccines by the FDA and the NMPA are inactivated, cell culture-based formulations that do not contain adjuvants. The standard route of administration is intramuscular injection, although certain vaccines are also approved for intradermal use, and they are indicated for all age groups. The principal distinction among these vaccines lies in their manufacturing platforms: While the FDA predominantly authorizes the Human Diploid Cell Rabies Vaccine (HDCV) and the Purified Chick Embryo Cell Vaccine (PCECV), with specific parameters such as dose and age indication detailed in Table 1, China also licenses vaccines produced in Vero cells and primary hamster kidney cells, whose comprehensive specifications are summarized in Table 2 [8,14,15]. The following section details the specific vaccines approved in each of these two regions:

## 3. Immunization Schedules of Currently Approved Rabies Vaccines

Based on the WHO position paper on rabies vaccines, management protocols are established for individuals of all age groups with different immune statuses (never immunized or previously immunized) following Category I, II, and III rabies exposures. These protocols specifically encompass wound/exposed skin cleansing requirements, the selection of PEP vaccination schedules, and recommendations for the use of rabies immune globulin (RIG) [8]. For details, see Table 3:

According to the Guidelines for the management of rabies exposure prophylaxis and treatment (2023 version) jointly issued by the National Disease Control and Prevention Administration & National Health Commission of the People’s Republic of China, standardized management protocols have been systematically defined for individuals with different immune statuses (first exposure, re-exposure, pre-exposure immunized populations) following Category I, II, and III rabies exposures. These protocols encompass core requirements such as wound management, rabies vaccination schedules, and the use of immune globulin [16]. For details, see Table 4:

## 4. Adjuvants

### 4.1. Immunomodulatory Molecule Adjuvants

#### 4.1.1. Bacterial Products

##### Monophosphoryl Lipid A (MPLA)

MPLA is a detoxified derivative of bacterial lipopolysaccharide (LPS). While the parent LPS molecule is a potent but highly toxic immune activator unsuitable for direct human or animal use, the removal of a phosphate group coupled with partial deacylation yields MPLA. This chemical modification significantly reduces toxicity while retaining potent adjuvant activity [17]. As a Toll-like receptor 4 (TLR4) agonist, MPLA activates innate immunity by engaging pattern recognition receptors (PRRs) [18].

Chen et al. [19] systematically examined the effect of combining the TLR4 agonist MPLA with an inactivated rabies vaccine on immune cell activation, antibody production, and protective efficacy. The study demonstrated that MPLA acts through a TLR4-dependent pathway to markedly enhance the maturation of bone marrow-derived dendritic cells (BMDCs) in vitro and effectively promotes the activation and maturation of conventional dendritic cells (cDCs) in the inguinal lymph nodes in vivo. Activated cDCs upregulated the expression of co-stimulatory molecules (CD80 and CD86) and MHC class II, thereby enhancing their interaction with T cells. Moreover, MPLA significantly increased the recruitment of follicular helper T cells (Tfh), the proliferation of germinal center B cells (GC B cells), and the generation of bone marrow plasma cells. These effects collectively promoted the production of rabies virus (RABV)-specific total IgG, IgG2a, IgG2b, and virus-neutralizing antibodies (VNA). Serological analysis revealed that in mice immunized with MPLA-adjuvanted vaccine, VNA titers remained at or above 0.5 IU/mL—the protective threshold defined by the WHO—both one week and eight weeks after immunization, indicating that MPLA not only accelerates the humoral response but also sustains long-term immunity. In a viral challenge experiment, all mice receiving the MPLA-adjuvanted vaccine survived a lethal dose (100× LD_50_) of the virulent DRV-Mexico strain, compared to only 50% survival in the group administered the inactivated vaccine alone. These results confirm the strong protective efficacy afforded by MPLA against pathogenic RABV infection.

Hu et al. [20] conducted a study using 6–8-week-old SPF BALB/c mice, which were randomly allocated into three groups: an MPLA–rabies vaccine complex group, a rabies vaccine-alone group, and a sterile saline control group (*n* = 16 per group). Mice were immunized twice via intraperitoneal injection at a one-week interval. Immune responses were evaluated using enzyme-linked immunosorbent assay (ELISA), lymphocyte transformation test (LTT), enzyme-linked immunospot (ELISPOT) assay, and flow cytometry. Compared with the vaccine-alone group, the MPLA-adjuvanted group not only accelerated the production of specific antibodies—inducing detectable antibodies 10 days earlier—but also elicited significantly higher antibody concentrations on days 14 and 21 post-immunization. On day 14, the antibody concentration in the MPLA group was nearly twice that of the vaccine-alone group (*p* < 0.05), supporting the rapid establishment of immune protection during the incubation period. At the cellular level, the LTT showed significantly enhanced splenic lymphocyte proliferation in the MPLA group (*p* < 0.05). ELISPOT analysis revealed a threefold increase in the number of cells secreting IFN-γ (a Th1 cytokine) and IL-4 (a Th2 cytokine) compared to the vaccine-alone group (*p* < 0.05), with a predominant Th2-type response. Flow cytometry further confirmed that MPLA significantly upregulated the activation of both CD3^+^CD4^+^ and CD3^+^CD8^+^ T cells (*p* < 0.05), with a more pronounced increase in CD3^+^CD4^+^ T cells. CD3^+^CD8^+^ cytotoxic T lymphocytes can directly participate in viral clearance, and the coordinated activity of these T-cell subsets contributes to a comprehensive antiviral immune defense.

##### Flagellin

Flagellin, the filamentous structural protein of bacterial flagella, exhibits strong inherent antigenicity. As a pathogen-associated molecular pattern (PAMP), it is recognized by Toll-like receptor 5 (TLR5) on host cells such as epithelial cells, monocytes, granulocytes, and dendritic cells. Binding of flagellin to TLR5 activates downstream signaling pathways, induces cytokine production, promotes inflammatory responses, and ultimately initiates specific adaptive immunity [21]. In a study by Xiao et al. [22], the adjuvant properties of three *Salmonella* Typhimurium flagellins—FljB, FliC, and the fusion protein FljB-FliC—were evaluated in combination with an inactivated whole killed rabies vaccine (WKRV) to enhance vaccine immunogenicity. In BALB/c mice, immunization with either FljB or FliC as an adjuvant significantly increased anti-rabies IgG antibody titers compared to vaccine alone. Notably, FljB induced an earlier onset of antibody production than FliC, enabling more timely immune protection. At the cellular level, both flagellins promoted splenocyte proliferation and significantly enhanced secretion of the cytokines IFN-γ and IL-4. Comparative analysis indicated that FljB elicited a stronger cellular immune response than FliC. Overall, based on its superior enhancement of both humoral and cellular immunity, FljB shows potential as an effective adjuvant for developing improved rabies vaccines with higher immunogenicity and protective efficacy.

##### CpG ODN

CpG oligodeoxynucleotides (CpG ODNs) are synthetic DNA sequences containing unmethylated cytosine–guanine motifs. They function as immune adjuvants by activating TLR9 on cells such as B cells and dendritic cells. This activation promotes a Th1-biased immune response, characterized by the secretion of cytokines including IL-12 and IFN-γ, and enhances antiviral as well as antitumor immunity [23]. Yu et al. [24] evaluated the adjuvant effect of a CpG ODN with cross-species immunostimulatory activity (active in both humans and mice) when combined with HDCV in a mouse model simulating human PEP. Compared to HDCV alone, co-administration with either 1.25 μg or 5 μg of CpG ODN accelerated the production of rabies virus-neutralizing antibodies (RVNA) and significantly improved seroconversion rates. On day 8 post-immunization, seroconversion reached 40% and 60% in the 1.25 μg and 5 μg CpG groups, respectively, whereas neither the HDCV-alone group nor a group receiving a high dose (20 μg) of CpG showed any seroconversion. By day 10, seroconversion in both lower-dose CpG groups increased to 100%, exceeding the 80% rate observed with HDCV alone. The 5 μg dose per mouse was identified as optimal. This regimen not only induced a geometric mean titer (GMT) of RVNA reaching 0.6 IU/mL by day 8 (significantly higher than HDCV alone, *p* < 0.05) but also maintained higher RVNA levels on days 10, 14, and 30. Furthermore, this dose significantly increased the number of IFN-γ-secreting splenocytes (approximately 500 SFU/5 × 10^5^ cells), indicating a stronger cellular immune response. Notably, even when the number of immunizations or the HDCV dose was reduced, the CpG-adjuvanted formulation still induced RVNA titers comparable to or higher than those achieved with the full-dose HDCV alone. Following challenge with CVS-11 virus (50 LD_50_), the survival rate of the full-dose HDCV–CpG group was 50%, while the survival rates of the half-dose and quarter-dose HDCV–CpG groups were both 40%. All these rates were higher than the 30% survival rate observed in the HDCV-alone group, demonstrating effective protection against rabies infection.

**Figure 1:**
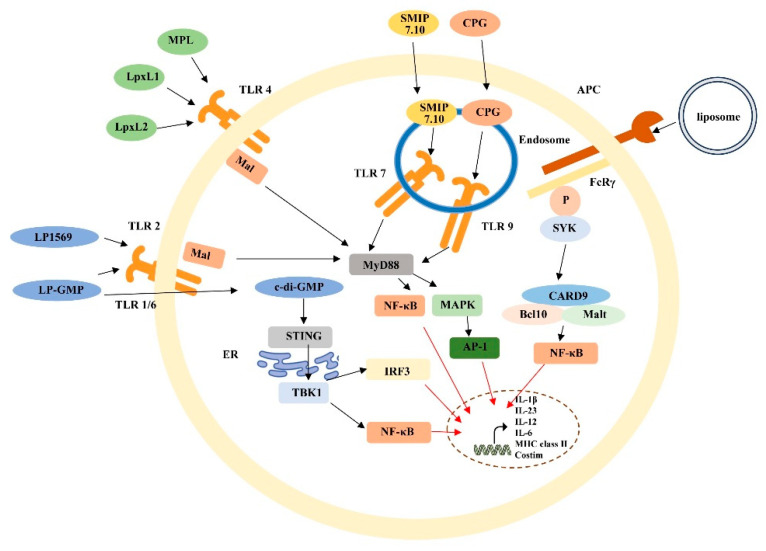
Schematic diagram illustrating that different types of adjuvants activate signaling pathways such as MyD88/NF-κB, prompting antigen-presenting cells to secrete cytokines like IL-12 and thereby preferentially inducing a Th1-type cellular immune response. (Reprinted from [25], licensed under CC BY 4.0.).

Different types of adjuvants program divergent adaptive immune responses by activating specific pattern recognition receptors. This mechanism is illustrated in Figure 1 [25]. As shown, novel adjuvants represented by MPL (a TLR4 agonist) and CpG ODN (a TLR9 agonist) can activate signaling pathways such as MyD88/NF-κB, driving antigen-presenting cells to secrete cytokines like IL-12, thereby preferentially inducing a Th1-type cellular immune response. In contrast, traditional alum adjuvants primarily promote a Th2-type humoral immune response.

#### 4.1.2. PIKA Adjuvant

The PIKA adjuvant is a chemically stabilized double-stranded RNA (dsRNA) analog that functions as a potent adjuvant [26]. It functions by activating innate immunity, thereby stimulating the production of cytokines including type I interferons (IFN-α, IFN-β), IFN-γ, interleukins (IL-2, IL-6, IL-12), and tumor necrosis factor-α (TNF-α). PIKA also promotes the maturation and activation of antigen-presenting cells (APCs), upregulating co-stimulatory molecules such as CD40, CD80, and CD86. This enhances both T-cell-mediated cellular immunity and humoral immune responses [27]. Zhang et al. [28] developed a novel rabies vaccine (PIKA-RV) using the TLR3 agonist PIKA as an adjuvant. In animal studies, this vaccine elicited rabies virus neutralizing antibody titers in mice nearly three times higher than those induced by the non-adjuvanted vaccine and significantly enhanced T-cell activation. In post-exposure protection models, protection rates reached 70% in beagle dogs and 67.7% in golden hamsters. When administered on an accelerated 7-day schedule, the protection rate in golden hamsters increased to 80%, compared to only 20–30% for the non-adjuvanted vaccine. Acute and repeated-dose toxicity studies in mice indicated that both PIKA and PIKA-RV were well-tolerated, supporting their potential as next-generation rabies vaccine candidates. Preliminary clinical trials have demonstrated favorable immunogenicity, tolerability, and safety of the PIKA-adjuvanted vaccine, meeting standards for prophylactic use [29]. Building on this, a pivotal Phase III trial is ongoing to further evaluate its immunogenicity, safety, and lot-to-lot consistency in a larger population [30].

#### 4.1.3. Plant-Derived Adjuvants

##### Isatis Root Polysaccharide

*Isatis indigotica* root polysaccharide (IIP) is a biologically active polysaccharide derived from the cruciferous plant *Isatis indigotica* (woad root) [31]. In one study [32], two distinct polysaccharides were isolated from Isatis root and evaluated as adjuvants in mice immunized with an inactivated rabies vaccine (rCVS-11-G), using aluminum hydroxide as a comparator. The study assessed neutralizing antibody titers, lymphocyte activation, cytokine secretion, challenge protection, and post-exposure immunization efficacy. Results demonstrated that both polysaccharides induced rabies virus-neutralizing antibodies (VNA) more rapidly and at higher titers than aluminum hydroxide. They also activated greater numbers of B and T lymphocytes in lymph nodes and blood, and enhanced splenocyte secretion of cytokines including IL-2, IL-4, IL-10, and IFN-γ. IIP-2 in particular showed superior efficacy, enhancing both humoral and cellular immunity simultaneously via Th1 and Th2 pathways. In challenge experiments, all mice receiving polysaccharide-adjuvanted vaccines survived. In post-exposure immunization, the survival rate in the IIP-2 group (7/10) was significantly higher than that in the aluminum hydroxide group. These findings indicate that *Isatis* root polysaccharides, especially IIP-2, are highly effective and safe adjuvants for rabies vaccines. They can improve immunogenic efficacy, accelerate antibody production, and reduce the required vaccine dose, showing promise for developing more economical human post-exposure rabies vaccines—particularly in developing countries.

##### Artesunate

Artesunate (ART) is an ester derivative of artemisinin, which is extracted from the medicinal plant *Artemisia annua*. Both artesunate and its derivative dihydroartemisinin (DHA) have been shown to inhibit rabies virus (RABV) replication at low concentrations. Studies indicate that ART and DHA can suppress viral genomic RNA synthesis and viral gene transcription, thereby inhibiting viral replication at the cellular level. Furthermore, ART and DHA have also exhibited certain therapeutic effects in animal studies, increasing the survival rate of mice challenged with the rabies virus [33]. In a study by Luo et al. [34], Kunming mice were used to evaluate the adjuvant potential of ART and DHA. It was first confirmed that intramuscular injection of ART or DHA at 5 mg/kg was non-toxic, with no observed weight loss or abnormal symptoms. The mice were then immunized with inactivated rabies virus strains CVS-11 or rHEP-dG (containing a double glycoprotein gene), each mixed separately with ART or DHA. Virus-neutralizing antibody (VNA) titers were measured using the fluorescent antibody virus neutralization (FAVN) test, followed by a lethal virus challenge. The results showed that ART significantly enhanced VNA levels induced by both vaccines, and the survival rate of immunized mice after challenge was markedly higher than that of the control group. In contrast, DHA did not demonstrate such an adjuvant effect. The authors speculate that this difference may be related to ART’s ability to modulate Th1/Th2 cytokine secretion and increase the number of regulatory T cells (Tregs), whereas DHA may bias the immune response by inhibiting IL-4 secretion. These findings suggest that ART is a safe and effective candidate adjuvant for rabies vaccines, with immunoenhancing effects that are independent of the viral strain. This work provides a valuable reference for the development of viral vaccine adjuvants and lays the groundwork for further exploration of other artemisinin derivatives.

##### Plant Saponins

Saponins from the Chilean soapbark tree (*Quillaja saponaria*), particularly the purified fraction QS-21, are potent immunostimulants widely used as vaccine adjuvants [35]. The crude bark extract contains a mixture of saponins, including QS-7, QS-17, QS-18, and QS-21. Among these, QS-18 is the most abundant but also the most toxic in mice, whereas QS-21 is less toxic and has become the most clinically relevant component due to its efficacy in licensed human vaccines [36]. Wang et al. [37] developed a broadly applicable protein–polymer nanoadjuvant platform by integrating the potent adjuvant QS-21 into an immunomodulatory nanocarrier (PPCD) composed of PLA-Porphyrin-Co^2+^ and DSPE-PEG_2000_, which self-assembles into micellar nanoparticles. In mice, this nanoparticle system combined with a recombinant rabies viral protein antigen elicited robust humoral immunity (high-titer neutralizing antibodies) and cellular immunity (activation of CD8^+^ T cells), effectively protecting against a lethal viral challenge.

Anna Carolina A. Yendo et al. [38] evaluated an aqueous leaf extract (AE) of *Quillaja brasiliensis* and its purified saponin fractions (QB-80, QB-90, Fraction B, Fraction 3) as rabies vaccine adjuvants. Compared with commercial adjuvants Quil-A and alum in a mouse model, the *Q. brasiliensis* saponins significantly enhanced specific antibody responses—inducing both Th1-type IgG2a and Th2-type IgG1—and promoted stronger cellular immunity. Both single- and two-dose regimens effectively protected mice against a lethal rabies virus (CVS strain) challenge, with no viral RNA detected in the brains of survivors. These saponins were also less toxic than Quil-A, and the use of leaves as a renewable resource offers an advantage over bark-derived extracts. The study supports their feasibility as alternative or complementary adjuvants for veterinary rabies vaccines, with potential for future human use.

Su et al. [39] evaluated ginsenoside Re as an adjuvant to enhance the immune response to an inactivated rabies vaccine (RV) in ICR mice. Groups included saline, Re alone, various RV doses, and RV combined with different Re concentrations. Assessments included serum rabies-specific antibody titers, splenocyte proliferation, cytokine mRNA expression (IL-4, IL-10, IL-12, IFN-γ), and proportions of peripheral blood CD4^+^ and CD8^+^ T cells. The combination of Re and RV significantly increased antibody titers, prolonged antibody persistence, raised the CD4^+^/CD8^+^ ratio, promoted lymphocyte proliferation, and activated both Th1 and Th2 responses (upregulating relevant cytokines). The immunogenicity achieved with 20 µL of RV plus 5.00 mg/kg Re was comparable to that of 100 µL RV alone. The study concluded that Re can effectively enhance the immunogenicity of inactivated rabies vaccine, potentially allowing dose reduction and lower vaccination costs.

##### Lentinan (LNT)

LNT, a β-1,3-glucan with β-1,6-branching structures derived from *Lentinula edodes*, is a clinically approved immunomodulator for cancer therapy in Japan [40]. Its application has recently expanded into vaccinology, where it demonstrates particular utility in single-dose, controlled-release formulations. A compelling example is its incorporation into a rabies virus glycoprotein (RVG) subunit vaccine. In this system, LNT and antigen were co-encapsulated in TA/PEG-coated CaCO_3_ microspheres engineered for pulsatile release, effectively mimicking a conventional multi-dose schedule (days 0, 3, 7, 14, and 28) with a single injection. Compared to non-adjuvanted controls, the LNT-adjuvanted vaccine elicited significantly higher neutralizing antibody titers and enhanced splenocyte proliferation. It also fostered a balanced Th1/Th2 cytokine profile, characterized by elevated levels of IFN-γ, TNF-α, IL-4, and IL-6. Notably, the vaccine achieved high in vitro viral inhibition, and its overall immunogenicity was comparable to a regimen requiring five separate injections. These results underscore LNT’s potential as an effective adjuvant for next-generation rabies vaccines. By enhancing antigen presentation and broad immune activation—likely through engagement of macrophages, dendritic cells, and lymphocytes—LNT-based controlled-release systems offer a pragmatic strategy to simplify post-exposure prophylaxis (PEP) and improve compliance [41].

#### 4.1.4. Cytokine Adjuvants

Cytokine-based adjuvants function by precisely modulating immune cell development and signaling pathways to augment antigen-specific responses. Interleukin-7, a central cytokine for lymphocyte homeostasis, has emerged as a particularly promising candidate in this class [42]. In an innovative approach, Wang et al. [42] leveraged this potential by co-encapsulating mRNA encoding the RVG and mRNA encoding IL-7 within lipid nanoparticles, creating a single-dose “G&IL-7 mRNA” vaccine. Compared to a control group receiving the antigen mRNA vaccine alone, this construct demonstrated a marked superiority in establishing long-term immunity. It elicited significantly higher titers of virus-neutralizing antibodies that remained above the WHO protective threshold of 0.5 IU/mL for at least six months. Mechanistically, the vaccine robustly enhanced a follicular helper T cell-dependent germinal center response, which underpinned the generation of memory B cells and bone marrow-resident plasma cells, thereby solidifying the foundation for durable immunological memory. In a lethal challenge with the rabies virus conducted 24 weeks post-immunization, the single-dose G&IL-7 mRNA vaccine conferred complete (100%) protection, a outcome starkly superior to the 70% survival rate observed in the antigen-only mRNA vaccine group. These collective findings validate the core role of IL-7 mRNA as an adjuvant in enabling potent and sustained immunity with a single administration, offering a novel strategic avenue to simplify post-exposure prophylaxis regimens.

Beyond IL-7 mRNA, the mRNA-based adjuvant platform itself represents a transformative emerging strategy for rabies vaccine development [43]. Leveraging the ability of mRNA to efficiently encode immunomodulatory molecules (e.g., cytokines, costimulatory molecules) or antigen targets (e.g., RVG), this platform offers unique advantages: rapid design and production, flexibility to combine multiple immunogens/adjuvants in a single formulation, and the potential to induce both high-titer neutralizing antibodies and durable cellular immunity. As a next-generation technology, mRNA-based adjuvanted vaccines are being explored to further simplify PEP (e.g., single-dose regimens) and address the poor compliance associated with long schedules [44,45]. However, key technical barriers—including mRNA stability, delivery efficiency (reliance on lipid nanoparticles), and scalability for low-resource settings—remain to be fully resolved [46].

### 4.2. Antigen Delivery System Adjuvants

#### 4.2.1. Aluminum Salt Adjuvants

Aluminum salt adjuvants primarily include aluminum phosphate, aluminum hydroxide, and aluminum potassium sulfate. Among these, aluminum hydroxide and aluminum phosphate are the most commonly used in practice. Aluminum hydroxide adjuvant is, in fact, an incompletely dehydrated form of Al(OH)_3_, specifically the crystalline aluminum oxyhydroxide, AlO(OH). Aluminum phosphate adjuvant consists of an amorphous aluminum hydroxyphosphate complex. These adjuvants function by adsorbing antigens onto their surfaces via electrostatic interactions and ligand exchange, resulting in antigen precipitation. This adsorption facilitates the slow release of the antigen at the injection site, prolonging its in vivo retention and thereby enhancing the immune response [47]. Aluminum adjuvants were among the earliest adjuvants incorporated into rabies vaccines. In 1965, China began developing an inactivated rabies vaccine cultured in primary hamster kidney cells, which included aluminum hydroxide as an adjuvant to enhance immunogenicity. However, the use of aluminum adjuvants was associated with a delayed immune response and slower production of neutralizing antibodies. Consequently, in 2005, the NMPA mandated the removal of aluminum hydroxide adjuvant to further improve the safety and efficacy profile of the vaccine.

#### 4.2.2. Liposomes

Liposomes are microscopic vesicles consisting of phospholipid bilayers capable of effectively encapsulating antigen or drug molecules, thereby enhancing their stability and delivery efficiency. As adjuvants, liposomes function by fusing with macrophage cell membranes to deliver vaccine antigens into the cytoplasm, which enhances macrophage phagocytosis and antigen-presenting capacity [48]. A study by Guo et al. [49] showed that the immunoprotective efficacy induced in mice by a liposome-adjuvanted rabies vaccine was twice that induced by the non-adjuvanted vaccine. Neutralizing antibody titers measured after three immunizations with the liposome-formulated vaccine were higher than those obtained after five immunizations with the adjuvant-free vaccine. In challenge experiments, the survival rate of mice receiving three doses of the liposome-adjuvanted vaccine also exceeded that of mice receiving five doses of the non-adjuvanted vaccine. These results indicate that liposome adjuvants can reduce the number of required vaccine injections while maintaining, or even improving, protective immunogenicity.

### 4.3. Composite Adjuvants

Composite adjuvants are immunoenhancing preparations formulated by combining one or more adjuvant substances with additional components. Typically, the immunoenhancing activity of individual adjuvants is often limited; therefore, combining multiple adjuvant components is employed to achieve a synergistic enhancement of the adjuvant effect. Liu Ze et al. [50] developed a novel nanoemulsion adjuvant called Golden03 for rabies vaccines, which contains squalene, coenzyme Q10, Tween 80, Span 80, and succinate-sodium citrate buffer (with higher coenzyme Q10 and succinic acid levels than the classic MF59 adjuvant) and targets succinate dehydrogenase (SDH) to enhance immune responses; BALB/c mice were immunized intramuscularly three times on days 1, 3, and 7, and results showed that Golden03 significantly accelerated rabies virus-neutralizing antibody production (reaching the WHO protective threshold of ≥0.5 IU/mL as early as day 5 with a peak of 0.74 IU/mL, compared to day 14 for the adjuvant-free and MF59-adjuvanted vaccines), maintained 100% IgG seroconversion at 1/4 vaccine dose (over half at 1/6 dose) while reducing the vaccine dose by three-quarters, induced four-fold more IFN-γ-secreting splenocytes, upregulated pro-inflammatory cytokines (IFN-γ, IL-1β, TNF-α) and CD8^+^ T cell expression, significantly enhanced mitochondrial SDH activity (2.647 U/mg), and achieved a 100% survival rate in mice challenged with 50 × LD_50_ of CVS-11 (outperforming the adjuvant-free group’s 12.5% and MF59 group’s 62.5% survival) with no obvious weight loss, indicating it enhances both humoral and cellular immunity and holds promise as an adjuvant for human rabies vaccines to address low immunogenicity, multiple injections, and high costs of current vaccines.

In addition to the Golden03 nanoemulsion adjuvant, a 2025 study by Cao et al. [51] reported a promising QS21 + CpG composite adjuvant system for rabies vaccines. This formulation combines QS21 (a saponin-derived adjuvant) with CpG oligodeoxynucleotides (a TLR9 agonist) and targets the RVG as the antigen. The synergistic mechanism of QS21 and CpG is key to its efficacy: QS21 activates B cells and promotes dendritic cell maturation, while CpG drives Th1 polarization and prolongs the survival of antibody-secreting cells. In Balb/c mouse models, the QS21 + CpG-adjuvanted vaccine showed superior humoral immunity—its RVG-specific IgG titer (2,918,400) was 3.08-fold higher than that of the LNP-mRNA vaccine group and 45.6-fold higher than the alum-adjuvanted group. Virus-neutralizing antibody (RVNA) titers reached 2246 IU/mL, far exceeding the WHO protective threshold of 0.5 IU/mL. For cellular immunity, the vaccine induced significant levels of IFN-γ-secreting splenocytes, IL-2, and IFN-γ-producing CD4+ T cells—moderate but sufficient for protection. Notably, both the QS21 + CpG and mRNA vaccine groups achieved 100% survival against a lethal 50LD_50_ CVS-11 challenge, outperforming the alum-adjuvanted group (70% survival). This QS21 + CpG combination addresses the limitations of single adjuvants, balancing strong humoral and cellular immunity. It offers a viable strategy for developing low-dose, short-schedule human rabies vaccines, especially for PEP requiring rapid protective responses.

## 5. Challenges in the Development of Adjuvanted Rabies Vaccines

The primary challenges in developing adjuvanted rabies vaccines lie in balancing immunoenhancement with safety. While adjuvants can reduce the antigen dose required and elicit stronger immune responses, most current research remains at the preclinical stage, lacking validation in human clinical trials. This gap hinders a comprehensive assessment of their safety profiles. Many novel adjuvants—particularly molecular ones—demonstrate significant efficacy in animal models. However, due to species-specific differences, their effects may not translate directly to humans, greatly increasing the complexity and uncertainty of development. Furthermore, any new vaccine must demonstrate a clear advantage over existing, continually optimized commercial vaccines across multiple dimensions to achieve widespread adoption. This includes not only efficacy, such as inducing high-titer neutralizing antibodies more rapidly, but also safety (e.g., reducing adverse reactions like fever), cost-effectiveness, and practicality of the vaccination schedule (e.g., reducing the number of doses). For instance, the 2025-published nucleoside-modified rabies mRNA vaccine [45] has completed systematic preclinical safety and immunogenicity validation in mouse and non-human primate models, and its study data fully supports the vaccine meeting Phase I clinical trial enrollment criteria—with preclinical data showing it can induce higher neutralizing antibody titers with a single dose—they still face potential challenges of higher adverse event rates (projected from preclinical immunostimulation profiles) and limited scalability in resource-limited settings due to reliance on specialized LNPs production and cold chain storage. In contrast, PIKA-adjuvanted vaccines have demonstrated favorable safety and compatibility with existing inactivated vaccine production lines in Phase II/III trials, though they require more large-population, long-term clinical data to confirm non-inferiority compared to licensed vaccines [29,30]. Ultimately, for any novel candidate to achieve global public health impact—especially in high-burden, resource-limited regions—it must aim to meet the standards of WHO prequalification. This process evaluates not only efficacy and safety but also affordability, thermostability, and the scalability of manufacturing under Good Manufacturing Practice standards. Navigating this pathway represents a critical, yet often underexplored, translational milestone beyond early-phase clinical success [52,53,54]. These requirements further compound the translational challenges for adjuvanted rabies vaccines. Research in this field must therefore strive to achieve a multidimensional balance: enhancing immunogenicity, ensuring safety, maintaining technological feasibility, and meeting real-world application needs. Beyond the challenges inherent to specific technological platforms, the successful translation of adjuvanted vaccines also faces real-world barriers rooted in public health and practical application.

Achieving broad accessibility and high cost-effectiveness is pivotal for successful translation. As a representative potent adjuvant, QS-21 faces exorbitant costs due to its complex plant extraction process and challenges in sustainable supply chains. This directly limits vaccine accessibility and large-scale application in resource-limited regions such as Asia and Africa. Although exploratory efforts to reduce costs—such as laboratory plant cultivation or engineered yeast production—are underway, the high price remains a major practical barrier to widespread adoption in the short term [55,56]. In contrast, RBI-4000, as a self-replicating RNA (srRNA) vaccine platform, offers a core advantage in eliciting exceptionally durable immune responses. A Phase I clinical trial published in 2025 demonstrated that the rabies virus neutralizing antibodies it induced remained above the protective threshold for 8 months, with an antibody decay half-life six times longer than that of traditional inactivated vaccines [57]. This “single-shot, long-lasting” characteristic holds promise for reducing the overall number of vaccination doses and long-term protection costs, offering a novel strategy to overcome the cost barriers associated with conventional adjuvants. Crucially, from a public health perspective, drastic dose reduction is more than a matter of convenience; it is a fundamental equity metric [58,59]. Simplifying the PEP regimen from multiple visits to a single dose could dramatically improve compliance in remote or underserved communities, where access to healthcare is limited. By directly addressing this key barrier to completion of treatment, platforms that enable ultra-durable immunity have the potential to deliver more equitable life-saving protection [57,58,59].

Beyond innovations within human vaccine platforms, lessons from veterinary medicine—where cost and ease of use are paramount—can also inform strategies to overcome these economic and accessibility barriers. Adjuvant development for veterinary rabies vaccines, in particular, offers valuable cross-disciplinary insights. A 2025 study identified oxidized mannan as a promising veterinary adjuvant, which was shown to induce a balanced Th1/Th2 immune response and improve protection rates in animal challenge models [60]. Its favorable attributes—including low toxicity, cost-effectiveness, and scalability—align closely with the core requirements for human adjuvants. This is particularly relevant for resource-limited settings, where high vaccination coverage in both populations is pivotal for effective disease control. Data from such veterinary adjuvant studies can inform human adjuvant development, providing references for optimizing immune balance and reducing production costs. This approach helps address the translational challenges of adjuvanted rabies vaccines from a broader prevention and control perspective.

## 6. Conclusions

In recent years, numerous studies have been reported on adjuvants for rabies vaccines. This article has reviewed the research progress on immunomodulatory molecular adjuvants, delivery-system adjuvants, and composite adjuvants in the context of rabies vaccination. Conventional rabies vaccines are associated with several limitations, including complex immunization schedules, delayed antibody production, low potency, and short-lived immunity. To address these issues, the development of novel rabies vaccine adjuvants should fulfill the following functions:

In terms of immune response, adjuvants should be capable of modulating or enhancing the antigen-specific immune reaction. Before the rabies virus invades the nervous system, the adjuvant should rapidly stimulate the production of high-titer specific neutralizing antibodies to promptly block viral infection of cells. Furthermore, it should induce a sufficiently strong immune response to allow for a reduction in the vaccine dose and a simplification of the immunization schedule, while also eliciting cellular immunity to clear already infected cells.

Additionally, vaccine adjuvants should exhibit minimal toxicity, and any potential adverse effects must be well-characterized to ensure safety during use. Finally, they should reduce the antigen demand of the vaccine, serving an antigen-sparing role.

Based on a thorough investigation of the physicochemical properties of antigens and adjuvants, their concentration ratios, and their mechanisms of interaction, we should continue to advance the research, development, and clinical translation of novel rabies vaccine adjuvants. Further efforts should be directed toward developing new adjuvants that are highly effective, low in toxicity, safe, and cost-effective.

## Figures and Tables

**Table 1:** Rabies Vaccines Approved by the FDA.

Vaccine Type	Adjuvant	Production Platform	Dose	Age Indication
Inactivated	None	Human diploid cell	≥2.5 IU/1 mL	All ages
Inactivated	None	Purified chick embryo cell	≥2.5 IU/1 mL	All ages

**Table 2:** Rabies Vaccines Approved by the NMPA (China).

Vaccine Type	Adjuvant	Production Platform	Dose	Age Indication
Inactivated	None	Human diploid cell	1 mL/dose (≥2.5 IU)	All ages
Inactivated	None	Purified chick embryo cell	1 mL/dose (≥2.5 IU)	All ages
Inactivated	None	Vero cell	0.5 mL/dose (≥2.5 IU)	All ages
Inactivated	None	Primary hamster kidney cell	1 mL/dose (≥2.5 IU)	All ages

**Table 3:** PEP by category of exposure [8].

Immunization Status	Category I Exposure	Category II Exposure	Category III Exposure
Immunologically naive individuals of all age groups	- Washing of exposed skin surfaces - No PEP required	- Wound washing and immediate vaccination: • 2-sites ID on days 0, 3 and 7 or • 1-site IM on days 0, 3, 7 and between day 14–28 or • 2-sites IM on day 0 and 1-site IM on days 7, 21 - RIG is not indicated	- Wound washing and immediate vaccination • 2-sites ID on days 0, 3 and 7 or • 1-site IM on days 0, 3, 7 and between day 14–28 or • 2-site IM on day 0 and 1-site IM on days 7, 21 - RIG administration is recommended
Previously immunized individuals of all age groups	- Washing of exposed skin surfaces - No PEP required	- Wound washing and immediate vaccination *: • 1-site ID on days 0 and 3 or • At 4-sites ID on day 0 or • At 1-site IM on days 0 and 3 - RIG is not indicated	- Wound washing and immediate vaccination *: • 1-site ID on days 0 and 3 or • At 4-sites ID on day 0 • At 1-site IM on days 0 and 3 - RIG is not indicated

* Immediate vaccination is not recommended if complete PEP already received within. ID: intradermal injection; IM: intramuscular injection; RIG: rabies immunoglobulins.

**Table 4:** Management Protocol for Rabies Exposure Based on Immune Status [16].

Immunization Status	Category I Exposure	Category II Exposure *	Category III Exposure
Primary Exposure (Incomplete full-course rabies vaccination)	- Clean the exposed skin - No vaccination required - No immunoglobulin required	- Thorough wound irrigation + disinfection - Rabies vaccination (choose one): • 5-dose schedule: 1 dose each on days 0, 3, 7, 14, 28 • “2-1-1” schedule: 2 doses on day 0, 1 dose each on days 7 and 21 - No immunoglobulin required	- Thorough wound irrigation + disinfection (debridement for penetrating wounds) - Rabies vaccination (same as primary Category II schedule) - Administration of rabies immunoglobulin (20 IU/kg, via wound infiltration injection)
Subsequent Exposure (Completed full-course rabies vaccination)	- Clean the exposed skin - No vaccination required - No immunoglobulin required	- Thorough wound irrigation + disinfection - <3 months after full vaccination: No vaccination required - ≥3 months after full vaccination: 1 dose each on days 0 and 3 - No immunoglobulin required	- Thorough wound irrigation + disinfection (debridement for penetrating wounds) - <3 months after full vaccination: No vaccination required - ≥3 months after full vaccination: 1 dose each on days 0 and 3 - No immunoglobulin required
Pre-exposure Immunized Individuals (Completed pre-exposure immunization schedule)	- Clean the exposed skin - No vaccination required - No immunoglobulin required	- Thorough wound irrigation + disinfection - <1 year after immunization: No vaccination required - 1–3 years after immunization: 1 dose on day 0 - ≥3 years after immunization: 1 dose each on days 0 and 3 - No immunoglobulin required	- Thorough wound irrigation + disinfection (debridement for penetrating wounds) - <1 year after immunization: No vaccination required - 1–3 years after immunization: 1 dose on day 0 - ≥3 years after immunization: 1 dose each on days 0 and 3 - No immunoglobulin required

* For cases confirmed as Category II exposure, if the exposed individual is severely immune-compromised, or if the wound site is on the head or face and the health status of the animal causing the injury cannot be determined, the case should be managed as a Category III exposure.

## Data Availability

No new data were created or analyzed in this study. Data sharing is not applicable to this article.

## References

[1] Klein R.S., Levin M.C. (2025). Rabies. In MSD Manual Professional Edition. https://www.msdmanuals.com/professional/neurologic-disorders/brain-infections/rabies.

[2] Schneider M.C., Sciancalepore S. (2025). Heading in the direction of ending human deaths from dog-mediated rabies. Lancet Infect. Dis..

[3] Tidman R., Thumbi S.M., Wallace R., De Balogh K., Iwar V., Dieuzy-Labaye I., Song J., Shadomy S., Qiu Y., Torres G. (2022). United Against Rabies Forum: The One Health Concept at Work. Front. Public Health.

[4] Quan N.K., Taylor-Robinson A.W. (2024). Combatting rabies outbreaks in Vietnam: High time to enforce restrictions on dog meat farming, a key source of transmission. IJID Reg..

[5] Müller T., Rupprecht C.C., Fooks A.R., Both L., Smith S.P., Gibson A.P., Lohr F., Fahrion A., Freuling C.M., Sing A. (2023). Elimination of Rabies: A Missed Opportunity. Zoonoses: Infections Affecting Humans and Animals.

[6] Riccardi N., Giacomelli A., Antonello R.M., Gobbi F., Angheben A. (2021). Rabies in Europe: An epidemiological and clinical update. Eur. J. Clin. Microbiol. Infect. Dis..

[7] Sparkes J., McLeod S., Ballard G., Fleming P.J.S., Körtner G., Brown W.Y. (2016). Rabies disease dynamics in naïve dog populations in Australia. Prev. Vet. Med..

[8] World Health Organization (2018). Rabies vaccines: WHO position paper. Wkly. Epidemiol. Rec..

[9] Ugolini G., Hemachudha T. (2018). Rabies: Changing prophylaxis and new insights in pathophysiology. Curr. Opin. Infect. Dis..

[10] Gluska S., Zahavi E.E., Chein M., Gradus T., Bauer A., Finke S., Perlson E. (2014). Rabies Virus Hijacks and accelerates the p75NTR retrograde axonal transport machinery. PLoS Pathog..

[11] Pattanaik A., Mani R.S. (2019). WHO’s new rabies recommendations: Implications for high incidence countries. Curr. Opin. Infect. Diseases.

[12] Bote K., Nadal D., Abela B. (2023). WHO’s latest rabies recommendations and guidance save lives and reduce the cost of treatment. One Health Implement. Res..

[13] Wang H., Wang S., Fang R., Li X., Xing J., Li Z., Song N. (2023). Enhancing TB Vaccine Efficacy: Current Progress on Vaccines, Adjuvants and Immunization Strategies. Vaccines.

[14] Centers for Disease Control and Prevention (2023). Human Rabies Prevention—United States, 2023 Recommendations of the Advisory Committee on Immunization Practices (ACIP). MMWR Recomm. Rep..

[15] Zhou X., Wu X., Cai Y., Cao S., Zhu X., Lv Q., Chen H., Shi L., Li J., Wang X. (2019). Pre-marketing immunogenicity and safety of a lyophilized purified human diploid cell rabies vaccine produced from microcarrier cultures: A randomized clinical trial. Hum. Vaccin. Immunother..

[16] National Disease Control and Prevention Administration & National Health Commission of the People’s Republic of China (2023). Guidelines for the Management of Rabies Exposure Prophylaxis and Treatment (2023 Version). https://cdcp.gd.gov.cn/zwgk/zcfg/flfg/content/post_4254940.html.

[17] Jiang Z.H., Budzynski W.A., Qiu D., Yalamati D., Koganty R.R. (2007). Monophosphoryl lipid A analogues with varying 3-O-substitution: Synthesis and potent adjuvant activity. Carbohydr. Res..

[18] Wang Y.Q., Bazin-Lee H., Evans J.T., Casella C.R., Mitchell T.C. (2020). MPL Adjuvant Contains Competitive Antagonists of Human TLR4. Front. Immunol..

[19] Chen C., Zhang C., Li R., Wang Z., Yuan Y., Li H., Fu Z., Zhou M., Zhao L. (2019). Monophosphoryl-Lipid A (MPLA) is an Efficacious Adjuvant for Inactivated Rabies Vaccines. Viruses.

[20] Hu X., Liu R., Zhu N. (2013). Enhancement of humoral and cellular immune responses by monophosphoryl lipid A (MPLA) as an adjuvant to the rabies vaccine in BALB/c mice. Immunobiology.

[21] Li X., Cao Y., Mou M., Li J., Huang S., Zhang E., Yan H., Yang J., Zhong M. (2023). Enhanced TLR5-dependent migration and activation of antigen-loaded airway dendritic cells by flagellin. J. Leukoc. Biol..

[22] Xiao X.X., Zhang Y., Liu J.X., Wei Q.L., Yin X.P. (2016). Immunoenhancement with flagellin as an adjuvant to whole-killed rabies vaccine in mice. Arch. Virol..

[23] Kayraklioglu N., Horuluoglu B., Klinman D.M. (2021). CpG Oligonucleotides as Vaccine Adjuvants. Methods Mol. Biol..

[24] Yu P., Yan J., Wu W., Tao X., Lu X., Liu S., Zhu W. (2018). A CpG oligodeoxynucleotide enhances the immune response to rabies vaccination in mice. Virol. J..

[25] Chasaide C.N., Mills K.H.G. (2020). Next-Generation Pertussis Vaccines Based on the Induction of Protective T Cells in the Respiratory Tract. Vaccines.

[26] Yu P., Liu Y., Tao X., He Y., Liu Q., Wang B., Zheng H., Zhang N., Bi S., Zhu W. (2023). Potential option for rabies post-exposure prophylaxis: New vaccine with PIKA adjuvant against diverse Chinese rabies strains. Vaccine.

[27] Lau Y.F., Tang L.H., Ooi E.E. (2009). A TLR3 ligand that exhibits potent inhibition of influenza virus replication and has strong adjuvant activity has the potential for dual applications in an influenza pandemic. Vaccine.

[28] Zhang Y., Zhang S., Li W., Hu Y., Zhao J., Liu F., Lin H., Liu Y., Wang L., Xu S. (2016). A novel rabies vaccine based-on toll-like receptor 3 (TLR3) agonist PIKA adjuvant exhibiting excellent safety and efficacy in animal studies. Virology.

[29] Kalimuddin S., Wijaya L., Chan Y.F.Z., Wong A.W.L., Oh H.M.L., Wang L.F., Kassim J.A., Zhao J., Shi Z., Low J.G. (2017). A phase II randomized study to determine the safety and immunogenicity of the novel PIKA rabies vaccine containing the PIKA adjuvant using an accelerated regimen. Vaccine.

[30] Yisheng Biopharma (Singapore) Pte. Ltd (2022–2023). A Phase III Study to Evaluate the Immunogenicity, Safety and Lot to Lot Consistency of Three Lots of a PIKA Rabies Vaccine (Vero cell) for Human Use, Freeze-Dried in Healthy Adults Using a Post-exposure Prophylaxis Schedule (NCT05667974). NCT05667974.

[31] Gao G., He C., Wang H., Guo J., Ke L., Zhou J., Chong P.H., Rao P. (2021). Polysaccharide Nanoparticles from Isatis indigotica Fort. Root Decoction: Diversity, Cytotoxicity, and Antiviral Activity. Nanomaterials.

[32] Zhang W., Zheng X., Cheng N., Gai W., Xue X., Wang Y., Gao Y., Shan J., Yang S., Xia X. (2016). Isatis indigotica root polysaccharides as adjuvants for an inactivated rabies virus vaccine. Int. J. Biol. Macromol..

[33] Luo J., Zhang Y., Wang Y., Liu Q., Li J., He H., Luo Y., Huang S., Guo X. (2021). Artesunate and Dihydroartemisinin Inhibit Rabies Virus Replication. Virol. Sin..

[34] Luo J., Zhang Y., He H., Liu Q., Huang S., Guo X. (2019). Artesunate enhances the immune response of rabies vaccine as an adjuvant. Vaccine.

[35] Martin L.B.B., Kikuchi S., Rejzek M., Owen C., Reed J., Orme A., Misra R.C., El-Demerdash A., Hill L., Hodgson H. (2024). Complete biosynthesis of QS-21 in engineered yeast. Nat. Chem. Biol..

[36] Bai D., Kim H., Wang P. (2024). Development of semisynthetic saponin immunostimulants. Med. Chem. Res..

[37] Wang C., Geng Y., Wang H., Ren Z., Hou Q., Fang A., Wu Q., Wu L., Shi X., Zhou M. (2024). A broadly applicable protein-polymer adjuvant system for antiviral vaccines. EMBO Mol. Med..

[38] Yendo A.C., de Costa F., Cibulski S.P., Teixeira T.F., Colling L.C., Mastrogiovanni M., Soulé S., Roehe P.M., Gosmann G., Ferreira F.A. (2016). A rabies vaccine adjuvanted with saponins from leaves of the soap tree (Quillaja brasiliensis) induces specific immune responses and protects against lethal challenge. Vaccine.

[39] Su X., Pei Z., Hu S. (2014). Ginsenoside Re as an adjuvant to enhance the immune response to the inactivated rabies virus vaccine in mice. Int. Immunopharmacol..

[40] Ina K., Kataoka T., Ando T. (2013). The use of lentinan for treating gastric cancer. Anticancer. Agents Med. Chem..

[41] Zhou X., Wang H., Zhang J., Guan Y., Zhang Y. (2024). Single-injection subunit vaccine for rabies prevention using lentinan as adjuvant. Int. J. Biol. Macromol..

[42] Wang L., Wan J., He W., Wang Z., Wu Q., Zhou M., Fu Z.F., Zhao L. (2024). IL-7 promotes mRNA vaccine-induced long-term immunity. J. Nanobiotechnol..

[43] Li D., Wang X., Li G., Zhou J., Bian L., Zhao X., Xing L., Zeng J., Cui J., Cui L. (2025). Optimizing rabies mRNA vaccine efficacy via RABV-G structural domain screening and heterologous prime-boost immunization. NPJ Vaccines.

[44] Li J., Liu Q., Liu J., Wu X., Lei Y., Li S., Zhao D., Li Z., Luo L., Peng S. (2022). An mRNA-based rabies vaccine induces strong protective immune responses in mice and dogs. Virol. J..

[45] Wang Y., Wang S., Huang L., Mao W., Li F., Lin A., Zhao W., Zeng X., Zhang Y., Yang D. (2025). A nucleoside-modified rabies mRNA vaccine induces long-lasting and comprehensive immune responses in mice and non-human primates. Mol. Ther..

[46] Pardi N., Krammer F. (2024). mRNA vaccines for infectious diseases—Advances, challenges and opportunities. Nat. Rev. Drug Discov..

[47] Shi S., Zhu H., Xia X., Liang Z., Ma X., Sun B. (2019). Vaccine adjuvants: Understanding the structure and mechanism of adjuvanticity. Vaccine.

[48] Tretiakova D.S., Vodovozova E.L. (2022). Liposomes as Adjuvants and Vaccine Delivery Systems. Biochem. (Mosc) Suppl. Ser. A Membr. Cell Biol..

[49] Guo X.X., Yan L., Yang Y., Yuan R., Liu Y., Sheng J. (2010). Immunization Schedule of Liposomal Rabies Vaccine in Animals. Chem. Res. Chin. Univ..

[50] Ze L., Zonglin L., Ya’Nan W., Shaohui S., Huijuan Y., Wei C., Li W., Liao G. (2019). Application of a novel nanoemulsion adjuvant for rabies vaccine which stabilizes a Krebs cycle intermediate (SDH) in an animal model. Hum. Vaccin. Immunother..

[51] Cao H., Li H., Liu W., Luan N., Hu J., Kong M., Song J., Liu C. (2025). A QS21+CpG-Adjuvanted Rabies Virus G Subunit Vaccine Elicits Superior Humoral and Moderate Cellular Immunity. Vaccines.

[52] World Health Organization (2013). WHO Expert Committee on Biological Standardization: Sixty-Fourth Report.

[53] World Health Organization (2024). Conditions for acceptance of an application. WHO Prequalification of Medical Products (IVDs, Medicines, Vaccines and Immunization Devices, Vector Control).

[54] Abela-Ridder B. Technical update on WHO’s position on rabies vaccines and post-exposure prophylaxis. Proceedings of the GAVI Human Rabies Vaccine Application Workshop.

[55] Liu Y., Zhao X., Gan F., Chen X., Deng K., Crowe S.A., Hudson G.A., Belcher M.S., Schmidt M., Astolfi M.C.T. (2024). Complete biosynthesis of QS-21 in engineered yeast. Nature.

[56] Son S.H., Kim J.E., Moon S.Y., Jang I.S., Yu B.J., Lee J.Y. (2022). Metabolic recycling of storage lipids promotes squalene biosynthesis in yeast. Biotechnol. Biofuels Bioprod..

[57] Maine C.J., Picarda G., Miyake-Stoner S.J., Essink B., Somodevilla G., Sparks J., Geall A.J., Wang N.S., Goldberg Z., Aliahmad P. (2025). Durability of next-generation self-replicating RNA vaccine RBI-4000: A phase 1, randomized open label clinical trial. Commun. Med..

[58] World Health Organization (2024). WHO position paper on rabies vaccines and immunoglobulins–October 2024. Wkly. Epidemiol. Rec..

[59] Dodman N. (2024). Zero by 30 and microarray patches. Lancet Reg. Health Southeast Asia.

[60] Mardani R., Bahmanje A., Kazeroni Y.C., Khoshroo F., Roshanaie B., Sadeghche T., Pajaie K., Hosseini S.N., Doroud D., Shahali M. (2025). Oxidized Mannan: A Novel Adjuvant Candidate for Enhancing Immune Responses in Veterinary Rabies Vaccine. Chonnam Med. J..

